# Understanding the Formation and Mechanism of Anticipatory Responses in *Escherichia coli*

**DOI:** 10.3390/ijms23115985

**Published:** 2022-05-26

**Authors:** Navneet Rai, Minseung Kim, Ilias Tagkopoulos

**Affiliations:** 1 UC Davis Genome Center, University of California-Davis, Davis, CA 95616, USA; nnrai@ucdavis.edu (N.R.); minseven77@gmail.com (M.K.); 2 Department of Computer Science, University of California-Davis, Davis, CA 95616, USA; 3 USDA/NSF AI Institute for Next Generation Food Systems (AIFS), University of California, Davis, CA 95616, USA

**Keywords:** anticipatory response, adaptation, genome-wide mutagenesis, catabolic genes, genetic network

## Abstract

Microorganisms often live in complex habitats, where changes in the environment are predictable, providing an opportunity for microorganisms to learn, anticipate the upcoming environmental changes and prepare in advance for better survival and growth. One such environment is the mammalian intestine, where the abundance of different carbon sources is spatially distributed. In this study, we identified seven spatially distributed carbon sources in the mammalian intestine and tested whether *Escherichia coli* exhibits phenotypes that are consistent with an anticipatory response given their spatial order and abundance within the mammalian intestine. Through RNA-Seq and RT-PCR validation measurements, we found that there was a 67% match in the expression patterns between the measured phenotypes and what would otherwise be expected in the case of anticipatory behavior, while 83% and 0% were in agreement with the homeostatic and random response, respectively. To understand the genetic and phenotypic basis of the discrepancies between the expected and measured anticipatory responses, we thoroughly investigated the discrepancy in D-galactose treatment and the expression of maltose operon in *E. coli*. Here, the expected anticipatory response, based on the spatial distribution of D-galactose and D-maltose, was that D-galactose should upregulate the maltose operon, but it was the opposite in experimental validation. We performed whole genome random mutagenesis and screening and identified *E. coli* strains with positive expression of maltose operon in D-galactose. Targeted Sanger sequencing and mutation repair identified that the mutations in the promoter region of *malT* and in the coding region of the *crp* gene were the factors responsible for the reversion in the association. Further, to identify why positive association in the D-galactose treatment and the expression of the maltose operon did not evolve naturally, fitness measurements were performed. Fitness experiments demonstrated that the fitness of *E. coli* strains with a positive association in the D-galactose treatment and the expression of the maltose operon was 12% to 20% lower than that of the wild type strain.

## 1. Introduction

Microbes live and evolve in dynamic ecosystems that differ widely in temperature, pH, osmotic stress and the availability of carbon sources [1,2]. These environmental changes can often occur at regular intervals or can have a predefined course of changes, providing an opportunity for a microbe to adapt and anticipate the upcoming condition based on the preceding signal or condition. An anticipatory response can help an organism make pertinent adaptive changes in advance to efficiently survive in a dynamic environment and hence may provide evolutionary and fitness advantages over organisms with a lower capability for adaptation. Several studies have demonstrated that bacteria have the potential to adapt and compete in dynamic and carbon source limited environments [3,4,5]. The mammalian digestive system, which has been inhabited by thousands of bacterial species for millions of years, is a perfect example of a dynamic ecosystem with spatial changes in stress, pH and metabolizable carbon sources. Recent studies suggest that the composition of gut microbiota influences the risk of several diseases in humans, including colorectal cancer, obesity, inflammatory bowel disease and type 2 diabetes. Moreover, it is now evident that dietary nutrients, including the nature and the availability of carbon sources for bacteria, have a significant impact on the composition and colonization of the gut microbiome [6,7].

Several carbon sources present in the diet have predefined spatial abundance in the intestine. According to the nutrient-niche theory, first developed by Freter [3,8], the nutrient landscape dictates which organisms can successfully colonize and persist in the gut [9]. Hence, it can be argued that the spatial abundance of different carbon sources in the human intestine provides an optimal platform for microbes, such as *Escherichia coli*, to develop correlative responses or even anticipate the upcoming environmental cue. *E. coli* is a well-studied model bacterium that travels back and forth between the mammalian intestine and the environment. Usually, *E. coli* is one of the first species to colonize the human intestine and inhabit there throughout human life [10]. In addition to the dynamic environment of the intestine, *E. coli* also faces competition from other bacterial species that inhabit the intestine [11]. Interestingly, only a handful of previous studies have suggested that *E. coli* has the potential to learn and anticipate the future environment. For example, anticipatory behavior in *E. coli* was first reported between the temperature upshift and changes in the oxygen level in the gut [12], and another study showed that D-lactose grown *E. coli* can anticipate the maltose [13]. More generally, the association between carbon sources and the genome-wide expression profile they induce has been investigated before in isolation [14]. It is still unknown how the correlation structure of the environment may be influencing anticipatory responses and the molecular mechanisms that facilitate these transitions.

**Figure 1:**
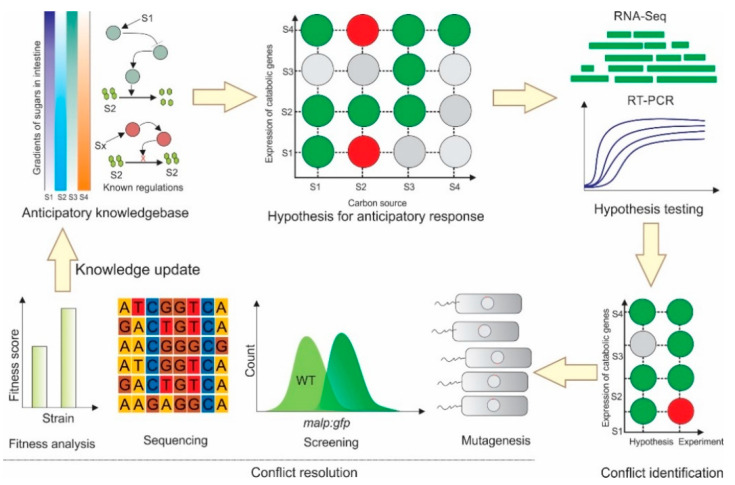
Overview of the hypothesis and validation of anticipatory responses in *E. coli* MG1655 for carbon sources that have spatial abundances in the mammalian intestine.

Here, we explored whether cross-dependencies and anticipatory responses exist in the expression profiles of *E. coli* cells exposed to the seven carbon sources (D-galactose, D-glucose, D-maltose, D-trehalose, D-fructose, D-lactose and Oleic acid) that have previously been reported to have concentration gradients in the mammalian intestine. *E. coli* was transcriptionally profiled after growth in these seven carbon sources, and differential expressions of the catabolic genes related to these seven carbon sources were calculated. We found 67% agreement between the measured anticipatory responses and what we would expect theoretically, while 83% and 0% of cases were in agreement with the homeostatic and random responses, respectively. To understand the genetic and phenotypic basis of the discrepancies between the expected and measured anticipatory responses, and to demonstrate if anticipatory responses can be rewired, as a case study, we thoroughly investigated the discrepancy in the D-galactose treatment and the expression of the maltose operon gene in *E. coli* through whole genome random mutagenesis, the screening of desired phenotypes, sequencing, and a cost-benefit analysis (Figure 1).

## 2. Results

### 2.1. Identification of Carbon Sources with Spatial Concentration Gradients in the Mammalian Intestine

**Figure 2:**
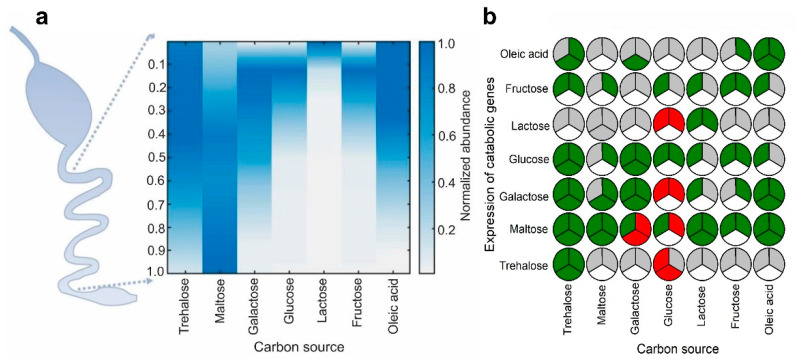
(**a**) The spatial abundance of different carbon sources in the intestine; (**b**) Expected and validated cross-regulation in the intestine. Top left slice, regulation based on the expectation and published knowledge; top right slice, regulation denoted from the RNA-Seq; bottom slice, regulation verified by the RT-PCR. Green, positive regulation; red, negative regulation; grey, no regulation; white, not measured.

A literature survey was performed to identify the carbon sources that have spatial abundances in the mammalian intestine. We were able to find a limited number of studies focusing on identifying the concentration gradients of carbon sources [15,16,17,18]. Based on these studies, we selected seven carbon sources (D-galactose, D-glucose, D-maltose, D-trehalose, D-fructose, D-lactose and Oleic acid) that have been reported to have spatial concentration gradients in the mammalian intestine and can be metabolized by *E. coli* (Figure 2a and Figure S1) in order to explore the existence of anticipatory responses in *E. coli* [15,16,17,18]. Since the data were collected from different sources, they had heterogeneity in the data points—hence, the reported concentrations of carbon sources and the length of the intestine were locally normalized from 0 to 1, and 1000 fitted data points for each carbon source were generated by Loess regression to obtain a better picture of spatial abundances (Figure 2a) [19]. In some cases, actual concentrations of the carbon sources were not reported in the literature; in such cases, either activities or concentrations of enzymes were used as proxies of the carbon source concentrations (Supplementary file 2). Figure 2a demonstrates the spatial concentration gradients of seven carbon sources across the mammalian intestine. For example, the concentration of D-lactose is high at the front end of the intestine, while that of D-maltose is high at the distal end of the intestine. As we mentioned earlier, bacteria such as *E. coli* have been passing through the mammalian intestine for millions of years and, in the standard case, will repeatedly experience a similar kind of spatial abundance as these seven carbon sources. So, we hypothesize that *E. coli*, which has an inherent capability to adapt to new conditions, may learn and anticipate the upcoming carbon sources, such as D-maltose, when it encounters an abundance of a particular carbon source, such as D-lactose, at the front end of the intestine. Based on these assumptions and the current knowledge, we created a map of the expected anticipation, demonstrating the presence of a carbon source and the expression of catabolic genes of relevant carbon sources (Figure 2b, top left slice). Subsequent transcriptome profiling and RT-PCR measurements were performed to check the validity of the expected anticipations.

### 2.2. Several Instances of Anticipatory Responses Were Identified at the Gene Expression Level in E. coli

Higher concentrations of carbon sources have been reported to exert metabolic pressure and reduce the growth of *E. coli* [20,21]. To identify the optimum concentration of the different carbon sources, we first measured the maximum growth rates (µ_max_) of *E. coli* at different concentrations of each of the seven carbon sources. All growth and subsequent experiments were always performed in M9 salt media supplemented with 0.1% glycerol as a background carbon source. Glycerol was chosen as a background carbon source because it has minimal influence on the carbon catabolite repression (CCR). CCR is a regulatory mechanism that regulates the sequential utilization of carbohydrates. CCR may interfere with the natural anticipatory response system [22,23,24]. Growth experiments were performed in 0.10% glycerol M9 supplemented with 5, 10, and 20 mM of each of the seven carbon sources mentioned above. It was observed that *E. coli* grown in a 10 mM concentration of each carbon source had consistently higher growth rates (Figure S2); hence, this concentration was chosen as the default in subsequent experiments. Later, the responses of *E. coli* at the transcriptome level were measured by performing RNA-Seq in 0.1% glycerol M9 supplemented with 10 mM of each of the seven carbon sources. Several cases of asymmetric and symmetric anticipatory responses were observed (Figure 2b), indicating the possibility of the existence of natural anticipatory responses. Out of 16 asymmetric and symmetric anticipatory responses, 69% were asymmetric and 31% were symmetric. Interestingly, in 80% of symmetric responses, carbon sources were inducing the expression of the catabolic genes of the second carbon source. The hypothesis that anticipatory responses can emerge through evolution in the gut was confirmed by the fact that 67% of the expected anticipatory profiles matched with the experimental anticipatory profiles obtained from the transcriptome profiling, while 83% and 0% agreed with the homeostatic and random response, respectively. We defined homeostatic response as when cells growing in a sugar induce or repress the carbon catabolic genes of sugars with a similar pattern of a concentration gradient in the intestine. We identified 18 such cases, and there were three differences between the expected response and the response measured using RNA-Seq. In random response, cells randomly induce the carbon catabolic genes of sugar with higher abundance in the upper part of the intestine while growing in sugar that has higher abundance in the lower part of the intestine. Three such cases were identified, and none of them matched the expectation. The findings of the transcriptome profiling were further validated by performing RT-PCRs of the targeted genes (Figure 2b and Figure S3) in selected conditions. There was 89% agreement between the findings of the transcriptome profiling and those of the RT-PCR.

### 2.3. Anticipatory Responses in E. coli Are Re-Programmable

**Figure 3:**
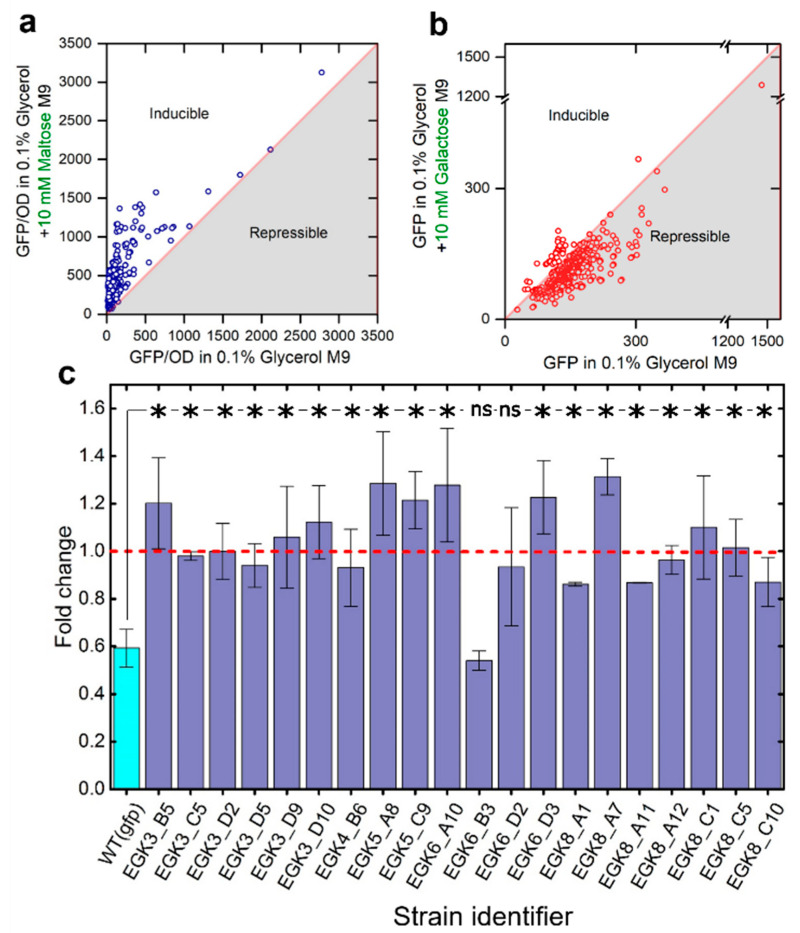
Screening of *E. coli* MG1655 mutants demonstrating a positive association between D-galactose treatment and the expression of *malP* (a gene of the maltose operon). (**a**,**b**) Screening of D-galactose and D-maltose responsive *E. coli* strains; (**c**) Further characterization of demonstrating a positive association between D-galactose treatment and the expression of *malP*, at least in triplicate. The responses reported in plot A were measured in a plate reader, and the rest of the measurements were taken using a flow cytometer. * indicates significant differences (*p*-value < 0.05), and ns indicates no significant differences (*p*-value > 0.05). The *p* values were calculated by the two-sample *t*-test. Error bars indicate the standard error of the mean (±).

**Figure 4:**
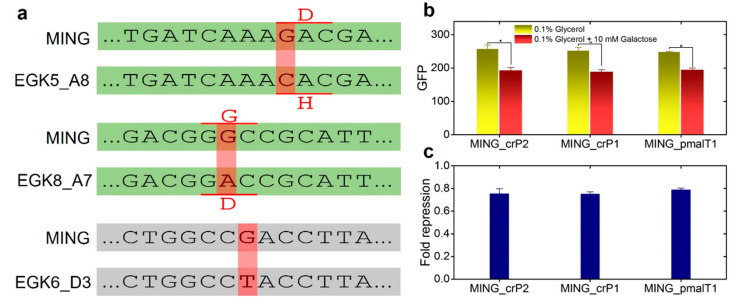
Identification of mutations and characterization of repair mutants. (**a**) Mutations in strains demonstrating the upregulation of *malP* expression in D-galactose supplemented media. EGK5_A8 and EGK8_A7 had point mutations in the coding region of *crP*, while EGK6_D3 had a point mutation in the promoter region of the *malt* gene; (**b**,**c**) Responses of repair mutants in 0.1% glycerol M9 and 0.1% glycerol M9 supplemented with 10 mM D-galactose. Fold repression was calculated by dividing the *gfp* (*malP*) levels in 0.1% glycerol M9 supplemented with 10 mM D-galactose with the *gfp* (*malP*) levels in 0.1% glycerol M9. Point mutations in EGK5_A8, EGK8_A7 and EG6_D3 were repaired to generate the repair mutants MING_crP2, MING_crP1 and MING_pmalT1, respectively. * indicates significant differences (*p*-value < 0.05). The *p* values were calculated by the two-sample t-test. Error bars indicate the standard error of the mean (±).

Next, we attempted to understand the mechanism of the formation of anticipatory behavior in *E. coli*. We picked up the case where D-galactose negatively regulates the expression of maltose operons and attempted to neutralize or reverse the association to understand the feasibility of reprogramming and the associated genetic and phenotypic consequences. The case of the negative anticipation of D-maltose once *E. coli* has encountered D-galactose is interesting, as it is the opposite of what *E. coli* should have evolved to do naturally in the intestine. In the mammalian intestine, the concentration of D-galactose is high at the front portion of the intestine, while D-maltose is high at the end of the intestine; hence, we can anticipate positive anticipation between D-galactose treatment and the expression of maltose operons because of millions of years of the adaptation of *E. coli* in the gut, but experimental observations indicate that *E. coli* shuts the expression of maltose operons when it encounters the D-galactose. To quantitate the expression of the maltose operon, we constructed *gfp:kanR* cassette and integrated that to downstream of the *malP* gene (maltose operon) in *E. coli* MG1655, generating the strain *E. coli* MING (Figure S3). We performed whole genome random mutagenesis in MING using ethyl methanesulfonate (EMS) to evolve the potential phenotypes demonstrating the induction of maltose operons (*malP:gfp*) in D-galactose supplemented growth media. EMS has been used widely for whole genome random mutagenesis to create the desired phenotypes in a short duration [25]. D-galactose and D-maltose inducible colonies were identified by measuring the *malP:gfp* expression separately in these carbon sources, as described in Section 4. Briefly, 455 colonies were screened (Figure 3a,b), and 31 colonies demonstrated a >1-fold increase in GFP fluorescence upon treatment with 10 mM D-galactose against the colonies grown in 0.1% glycerol. A total of 20 colonies demonstrating maximum fold change were further validated by characterizing them at least in triplicate in 10 mM D-galactose (Figure 3c). The colonies showing a positive response to the D-galactose treatment were Sanger sequenced. The potential genes and promoters that possibly influence the dose–response curve of the maltose operon (mal)—specifically the genes *malP*, *crP*, *DNA pol III*, *galR* and *malt* and the promoter regions of *malt*—were sequenced. The majority of mutants responding positively to D-galactose treatment did not have mutations in these genes, but we identified two *E. coli* mutants, EGK5_A8 and EGK8_A7, with unique mutations in the *crP* gene and one mutant, EGK6_D3, with the mutation in the promoter region of *malT* (Figure 4a). These observations indicate that the genetic networks of *E. coli* have the capability to create a positive association between D-galactose treatment and the expression of maltose operon.

#### Repair Mutants Reverse the Response to D-Galactose

To establish that the observed mutations in the EGK5_A8, EGK8_A7 and EGK6_D3 mutations are indeed responsible for the development of positive anticipatory responses between D-galactose treatment and the expression of *malP* gene (maltose operon), the mutations were fixed using the λ-red recombinase system [26] to generate the strains MING_crP2, MING_crP1 and MING_pmalT1, respectively. Sanger sequencing was performed to validate the fixation of the mutations. The responses of the repair mutants were measured in D-galactose by the flow cytometer, and, surprisingly, the repair mutants responded similarly to the parental *E. coli* strain MING, where the expression from the maltose operon (*malP:gfp*) was repressed in the 10 mM D-galactose supplemented M9 (Figure 4b).

### 2.4. Expression of Maltose Operons in D-Galactose Is Costly

**Figure 5:**
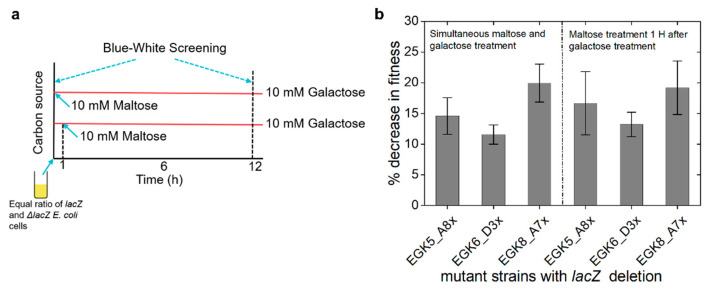
Competition assays to measure the fitness of *lacZ* deleted mutant strains showing a positive association between D-galactose treatment and *malP* expression. (**a**) Experimental strategy; (**b**) Fitness of *lacZ* deleted mutant strains against wild type strain MING.

We further investigated, given the feasibility of the emergence of a positive anticipatory response between D-galactose treatment and the expression of maltose operon, why the genetic networks of *E. coli* have not evolved to exhibit such phenotype, despite millions of years of encounters with high D-galactose at the top section and with high D-maltose at the bottom section of the intestine. To solve this puzzle, we first created the *lacZ* deleted strains of EGK5_A8, EGK8_A7 and EGK6_D3 (coined as EGK5_A8x, EGK8_A7x and EGK6_D3x), which competed against the parental strain MING in 0.1% glycerol M9 media, where either 10 mM each of D-galactose and D-maltose was provided simultaneously or where D-maltose was provided 1 h after D-galactose treatment (Figure 5a). These two time points were chosen between D-galactose and D-maltose treatment to determine the best time to see the conditioning effect of D-galactose. The quantification of the competition assay was conducted by growing treated cultures on LB agar plates supplemented with the IPTG and X-gal and then counting the blue/white colonies. It was observed that the fitness of the evolved mutant strains was 12% to 20% lower compared to that of the wild type strain when conditioned in the D-galactose, and the simultaneous experiments and those after 1 h of treatment with D-maltose showed similar responses (Figure 5b). These observations indicate that the cost of expressing maltose operons in D-galactose growth media is higher compared to the benefit; hence, *E. coli* has not evolved a positive association between D-galactose treatment and the expression of maltose operons.

## 3. Discussion

Environmental conditions are dynamic, and, in several instances, these conditions follow a specific course where, based on the current condition, the upcoming condition can be anticipated. An organism with the capability of learning and anticipation can prepare itself in advance to cope with the upcoming condition and hence will have the advantage of survival and growth over other organisms. Learning and anticipatory behaviors in mammals are well-studied phenomena, by which they are capable of associating two different environmental signals after repeated training. Pavlovian conditioning is a well-known example. Mammalian systems learn and anticipate primarily by making appropriate changes in the interconnections of neurons [27], but in microbes, such as *E. coli*, neurons are lacking, so any adaptive response generating anticipation should happen preferably through genetic and/or epigenetic modifications.

The environmental conditions of the mammalian intestine, though dynamic, harbor a sequential presence of predefined environmental composition. *E. coli* has been passing through the intestine for millions of years and hence has been trained indirectly to associate and anticipate the future environment based on the preceding environment. Only two studies have reported the existence of anticipatory behavior in *E. coli* [12,13]. Here, we explored the existence of anticipatory behavior in *E. coli* for seven carbon sources that have been reported to have spatial concentration gradients across the intestine. Surprisingly, we found only a limited number of studies demonstrating the concentration gradients of carbon sources. Based on evolutionary training and the current knowledge, a map of cross carbon source anticipatory behavior was created and validated experimentally, which was agreed upon at a rate of 67%. Different factors can contribute to the discrepancies, such as inaccuracies in our limited knowledge of the concentration gradients of carbon sources and the high cost of expressing catabolic genes for a second carbon source in advance. In one of the cases of disagreement, as per the expectation based on evolutionary training, there should be a positive correlation between the presence of D-galactose and the expression of maltose operons, but a negative correlation was observed. Whole genome random mutagenesis and selected screening demonstrated that *E. coli* have the capacity to upregulate the expression of maltose operons in D-galactose. The subsequent investigation revealed that, though *E. coli* has the capacity to upregulate maltose operons in D-galactose, due to the high cost of expression, it does not do so. These findings indicate that *E. coli* can anticipate the subsequent environmental conditions, and based on appropriateness, it can either create a positive or negative anticipatory response. This work can be further extended to study the existence and dynamics of anticipatory responses in the gut microbiota by performing metatranscriptomics in different carbon sources. Additionally, the selected microbial species can be trained to perform a specific task. For example, *E. coli* can be evolved and trained to secrete species-specific toxins when it encounters the targeted pathogenic bacteria in the intestine

## 4. Materials and Methods

### 4.1. Strains and Media

*E. coli* MG1655 [28] was used as a parental strain, and the strains derived from the parental strain are mentioned in Table 1. All *E. coli* strains were maintained at 4 °C on LB agar plates supplemented with the required antibiotic. For all the quantitative measurements, the cells were first grown overnight in LB broth. A fraction of the cells were transferred to a fresh M9 salt medium supplemented with 0.1% glycerol (Affymetrix) and grown for 8 h at 37 °C in an incubator shaker. Later, a fraction of the cells were transferred to the required media for either growth measurements or fluorescence measurements. All experiments were performed in three biological replicates unless otherwise indicated.

### 4.2. Growth Measurements at Different Concentrations of Carbon Sources

Fresh colonies of *E. coli* MG1655 were transferred to 1 mL LB broth media and grown for 8 h at 37 °C in an incubator shaker. After 8 h, 5 µL of grown cultures were transferred to 96 well plates (Costar) containing 195 µL of 0.1% glycerol M9 media supplemented with 5, 10, and 20 mM of carbon sources: D-galactose (Acros Organics, Geel, Belgium), D-Glucose (Acros Organics), D-maltose (Acros Organics), D-Trehalose (Fisher Scientific, Waltham, MA, USA), Oleic acid (Fisher Scientific) and D-Fructose (Acros Organics). Cell density (OD_600_) was measured every 15 min using a plate reader (BioTek HTX) at 37 °C for 24 h. The maximum growth rate (µ_max_) was calculated using the custom program in MATLAB^TM^. A total of 500 mM of a stock solution of oleic acid was prepared using water, Brij35 (Sigma, Burlington, MA, USA) and ethanol, while 500 mM of the stock solutions of the other five carbon sources was prepared in water. The stock solutions were filter sterilized using 0.22 µm PVDF filters (Olympus plastics).

### 4.3. Treatment of E. coli MG1655 with Carbon Sources, RNA Isolation and Transcriptome Profiling

Fresh colonies of *E. coli* were grown overnight in 0.1% glycerol M9. The next day, 50 µL of cells were transferred to 3 mL 0.1% glycerol M9 supplemented with or without 10 mM of each carbon source mentioned earlier and was grown and harvested in a mid log phase at 8 h. Then, 1 mL of the growing culture was mixed with the chilled 0.5 mL 5% phenol/ethanol (*v*/*v*), and the cells were subsequently pelleted down at 13,200 rpm at 4 °C. The supernatant was discarded carefully, and the cell pellets were stored immediately at −80 °C until use. RNA was extracted from the frozen cell pellet using a RNeasy kit (Qiagen, Hilden, Germany). The traces of any leftover genomic DNA were cured using an RNase-Free DNase Set (Qiagen). The concentration of total RNA was measured using a NanoDrop spectrophotometer (Thermo Fisher Scientific, Waltham, MA, USA). The quality of the isolated total RNA was checked by running 2 to 3 µg of total RNA on 1.5% denaturing agarose gel. For transcriptome profiling, 5 µg of total mRNA was taken, and mRNA enrichment was performed using a MICROB*Express*™ Bacterial mRNA Enrichment Kit (Thermo Fisher Scientific). The libraries from the mRNA enriched samples were prepared using the KAPA standard RNA-Seq library preparation kit (Kapa Biosystems, Wilmington, MA, USA). Double size selections (200 to 500 bp) of the libraries were performed using Agencourt AMPure XP (Beckman Coulter, Brea, CA, USA) beads. The quantification of the libraries was performed using a Qubit fluorometer (Thermo Fisher Scientific). The quality of the individual library was checked on a Bioanalyzer (Agilent Technologies, Santa Clara, CA, USA). The concentrations of all the libraries were normalized, and the libraries were pooled together and sequenced by an Illumina Hiseq 2500 system running in high throughput mode (Single end, 50 cycles). Since the minimum lengths of the RNA-Seq libraries were 200 bp, most of the tRNAs that are less than 100 nucleotides long [29] were lost during the fragmentation and size selection steps of the library preparation; hence, the tRNAs were excluded in the subsequent analysis. Please refer to Supplementary File 1 for a detailed analysis of the RNA-Seq data and the differentially expressed genes (DEGs).

### 4.4. Real-Time Reverse Transcription PCR (RT-PCR)

The cell growth, the treatment with specific carbon sources, the total RNA isolation and removal of genomic DNA were performed as described in the previous section. The quality of the isolated RNA was checked on 1.5% denaturing agarose gel. The first strand of cDNA was synthesized from 1 µg of RNA using a RevertAid™ First Strand cDNA Synthesis Kit (Thermo Scientific). The supplied random hexamers were used as primers. Gene expression was measured using the VeriQuest SYBR Green qPCR Master Mix on the ABI Viia 7 RT PCR platform (Thermo Scientific). A total of 2 µL of cDNA was mixed with 1 µL each of the required forward and reverse primers (10 µM; Table S1). The other components of the reaction mixtures were added as per the manufacturer’s instructions. The total reaction volume was made to 20 µL in 396 well plates. The following program was used for the amplification and quantification: 95 °C for 2 min, 40 cycles of 94 °C at 15 s, 60 °C for 1 min, 72 °C for 30 s, final annealing at 72 °C for 1 min, and melt curve from 55 °C to 95 °C for 20 min. The constitutive transcript *ihfb* was used as an internal control to normalize the expression [30].

### 4.5. Chromosomal Integration of gfp:kanR Cassette at the Downstream of malP

To directly measure the expression of the *malP* promoter, the *gfp:kanR* polycistronic cassette was integrated downstream of the chromosomal *malP* gene of *E. coli*. The *gfp* was amplified from BioBrick BBa_E0020 (parts.igem.org/Part:Bba_E0020) using the primers Gfp_in_fw and Gfpkan_rv1, while kanamycin was amplified from the plasmid psb3k3 (parts.igem.org/Part:pSB3K3) using the primers Kan_in_fw and Kan_gfp_rv (Table S2). Phusion polymerase (Thermo Fisher Scientific) was used for the error free PCR. The 3′ end of the primers Gfpkan_rv1 and Kan_in_fw had the restriction site for the XbaI. Kan_in_fw also had the ribosome binding site (BioBrick B0030) downstream of the XbaI site. After PCR amplification, the fragments were gel purified using a gel extraction kit (Thermo Fisher Scientific). Subsequently, the fragments were digested with XbaI for 3 h at 37 °C and later column purified. The purified fragments were ligated overnight at 16 °C using T4 ligase (NEB). A total of 5 µL of the ligated product was enriched by performing PCR using the primers Gfp_in_fw, Kan_gfp_rv, and Phusion polymerase. The PCR amplified products were gel eluted. The PCR amplified product was inserted downstream of the *malP* gene using the lambda red recombinase system, as described elsewhere [26]. The screening of the successful strain was performed by performing selection on LB agar plates containing 50 µg/mL kanamycin, and, later, the sequences and the site of insertion were verified by the Sanger sequencing of the *gfp:kanR* cassette and flanking regions.

### 4.6. Ethyl Methanesulfonate (EMS) Driven Random Mutagenesis and Screening of the Desired Mutant

A fresh colony of *E. coli*-MING was grown overnight at 37 °C in 20 mL LB broth supplemented with 50 µg/mL kanamycin. *E. coli* culture was washed with PBS (Phosphate Buffered Saline; 137.93 mM NaCl, 2.67 mM KCl, 1.47 mM KH_2_PO_4_, 8.1 mM Na_2_HPO_4_; pH of 7.4) at 8000 rpm for 2 min; then, the pellet was resuspended in PBS to the original volume. A total of 40 μL of EMS (ACROS Organics) was added to 2 mL of *E. coli* suspension, and the cells were incubated for 30, 60 and 120 min at 37 °C in an incubator shaker. After incubation, the cells were washed twice with PBS at 8000 rpm for 2 min. Then, they were resuspended in 2 mL of LB broth, grown for 1 h at 37 °C, serially diluted, and plated on LB agar plates. The LB agar plates were incubated overnight at 37 °C, and the grown colonies were used to screen the desired mutants. Individual bacterial colonies were grown overnight in 200 µL LB broth supplemented with 50 µg/mL kanamycin in 96 well plates at 37 °C in a plate reader cum shaker operating at 37 °C. The next day, 5 µL of grown culture was transferred to 195 µL of an M9 salt medium supplemented with 0.1% glycerol and 0.1% glycerol plus 10 mm D-maltose in 96 well plates. The cells were grown for 12 h in a plate reader at 37 °C, and the cell density (OD_600_) and GFP fluorescence (excitation: 485/20 nm, emission: 528/20 nm) were measured. After 12 h, 10 µL of the cultures grown only in 0.1% glycerol M9 was transferred to 190 µL of the M9 salt medium supplemented with 0.1% glycerol and both with 0.1% glycerol and 10 mM D-galactose. The cells were grown for 10 h in a plate reader at 37 °C. Later, the expressions of *gfp* in the individual cells were measured using a BD Accuri™ C6 cytometer (BD Biosciences). At least 20,000 events were recorded for each sample. For each event, forward- and side-scatter, as well as GFP levels (488 nm excitation laser; FL1 filter set), were recorded. The cells were selected from a tight forward and side-scatter gate to calculate the levels of GFP.

### 4.7. Mutation Repair

The repairs of the mutants were performed in two steps using the lambda red recombination system, as described in the above section. In the first step, the mutant genes were replaced by a spectinomycin resistance expressing cassette, and the subsequently integrated spectinomycin cassette was replaced with a cassette comprised of the wild type gene and the autonomously expressing chloramphenicol resistance gene. The integration was confirmed by Sanger sequencing. The spectinomycin cassette was amplified from the genomic DNA of *E. coli* MG1655Z1 [31] using the following primer sets: (i) SP7.2_FW and SPU_Rv to integrate the purified PCR product at the *crp* site of *E. coli* MING_crp1; (ii) SP7.4_Fw and SPU_Rv to integrate the purified PCR product at the *crp* site of *E. coli* MING_crp2; (iii) MT1S_Fw and MT1S_Rv to integrate the purified PCR product at the *malT* site of *E. coli* MING_malT (Table S3). All the primers had a 5′ 36- to 50-nucleotide region with perfect homology with their respective chromosomal integration sites. To facilitate the chromosomal integration, the *E. coli* strains MING_crp1, MING_crp2 and MING_malT were transformed with ampicillin resistance and the temperature sensitive plasmid pKD46 using the CaCl_2_ mediated heat shock method. The successful transformants were selected on an LB agar plate supplemented with 100 µg/mL ampicillin at 30 °C. The cells with plasmid pKD46 were made electrocompetent and transformed with respective spectinomycin cassettes using the BioRad electroporator using a 1mm cuvette at 1.75 KV. The successful transformants were selected on kanamycin (50 µg/mL) and spectinomycin (50 µg/mL) LB agar plates at 37 °C. Later, the integration was confirmed with PCR and Sanger sequencing. The primers used during various steps of the integration are mentioned in Supplementary Table S3.

### 4.8. Competition Assays

First, the *lacZ* gene of the mutant strains EGK5_A8, EGK8_A7 and EGK6_D3 was replaced by the spectinomycin resistance gene using the lambda red recombination system, as mentioned in the above sections, to generate the following Δ*lacZ* strains: EGK5_A8, EGK8_A7 and EGK6_D3, respectively. The competition assay was performed between wild type *E. coli* MG1655 and EGK5_A8, EGK8_A7 and EGK6_D3. All the *E. coli* strains were grown for 8 h in 3 mL LB broth at 37 °C in an incubator shaker; then, 50 µL of grown cultures were transferred to 2 mL of an M9 medium supplemented with 0.1% glycerol and was grown overnight at 37 °C in an incubator shaker. The cells were pelleted by centrifuging at 6000 rpm for 3 min. The supernatant was discarded, and the pellets were dissolved in 2 mL of the M9 salt medium. The OD_600_ was measured and normalized to the identical cell density using the M9 salt medium. The MG1655 was mixed separately with EGK5_A8, EGK8_A7, and EGK6_D3 in equal volume. Then, the competition assay was performed using two different protocols (Figure 5a): (i) 5 µL of the individual mixture of cultures was transferred to 195 µL of M9 media containing 0.1% glycerol, 10 mM D-galactose and 10 mM D-galactose in 96 well plates, and (ii) 5 µL of the individual mixture of cultures was transferred to 195 µL of M9 media containing 0.1% glycerol and 10 mM D-galactose in 96 well plates. The cells were grown for 1 h and then 10 mM D-maltose was added. The cells were grown, in total, for 12 h at 37 °C in a plate reader (BioTek HTX). The samples were collected at time 0 (start of plate reader) and at 12 h (completion of plate reader). The competition assay was performed with at least four biological replicates. The counting of the blue and white colonies was performed by growing the cells on LB agar plates containing 0.25 mM IPTG (Isopropyl-β-d-1-thiogalactopyranoside) and 40 mg/mL X-gal (bromo-chloro-indolylgalactopyranoside). The plates were incubated overnight at 37 °C and subsequently, the blue and white colonies were counted. The percentage loss in the fitness of the mutant strains against the wild type strain was calculated using the following equation:

$$x={\left[\frac{blue\;cfu}{white\;cfu}\right]}_{t=0},\;y={\left[\frac{blue\;cfu}{white\;cfu}\right]}_{t=12},\;\;Percentage\;loss\;in\;fitness=\left[\frac{y-x}{x}\right]\times 100$$

### 4.9. Statistical Analysis

All the data have been reported as the mean with the standard error. The values of the mean and standard errors were calculated from at least three separate experiments using the OriginPro software (OriginLab Corporation). The *p*-values were calculated by the two-sample *t*-test using the OriginPro software. The statistical analysis used in the analysis of the RNA-Seq data has been described in Supplementary File 1 (section, “Transcriptome analysis of RNA-Seq experiments” and “DEG (Differentially Expressed Gene) analysis”).

## Figures and Tables

**Table 1:** Strains used in this study.

Strain	Description	Reference
*E. coli* MG1655	Wild-type	Laboratory stock
MING	MG1655 (*malP:gfp:kan^R^*)	This study
EGK5_A8	MING with a point mutation in the coding region of *crP*	This study
EGK8_A7	MING with a point mutation in the coding region of *crP*	This study
EGK6_D3	MING with a point mutation in the promoter region of *malT*	This study
MING_crp1	EGK8_A7 with a repaired point mutation in the coding region of *crP*	This study
MING_crp2	EGK5_A8 with a repaired point mutation in the coding region of *crP*	This study
MING_pmalT1	EG6_D3 with a repaired point mutation in the promoter region of *malT.*	This study
EGK5_A8x	EGK5_A8 (Δ*lacZ*)	This study
EGK8_A7x	EGK8_A7x (Δ*lacZ*)	This study
EGK6_D3x	EGK6_D3x (Δ*lacZ*)	This study

## Data Availability

Data used to generate Figure 2a are available in the article (Supplementary file 2). Other data presented in this study are available on request from the corresponding author.

## Supplementary Materials

The following are available online at https://www.mdpi.com/article/10.3390/ijms23115985/s1.

## References

[1] Hughes B.S., Cullum A.J., Bennett A.F. (2007). An Experimental Evolutionary Study on Adaptation to Temporally Fluctuating pH in Escherichia coli. Physiol. Biochem. Zool..

[2] Roszak D.B., Colwell R.R. (1987). Survival strategies of bacteria in the natural environment. Microbiol. Rev..

[3] Freter R., Brickner H., Botney M., Cleven D., Aranki A. (1983). Mechanisms That Control Bacterial Populations in Continuous-Flow Culture Models of Mouse Large Intestinal Flora. Infect. Immun..

[4] Faith J.J., McNulty N.P., Rey F.E., Gordon J.I. (2011). Predicting a human gut microbiota’s response to diet in gnotobiotic mice. Science.

[5] Cooper V.S., Lenski R.E. (2000). The population genetics of ecological specialization in evolving *Escherichia coli* populations. Nature.

[6] Eetemadi A., Rai N., Pereira B.M.P., Kim M., Schmitz H., Tagkopoulos I. (2020). The Computational Diet: A Review of Computational Methods Across Diet, Microbiome, and Health. Front. Microbiol..

[7] Fan Y., Pedersen O. (2021). Gut microbiota in human metabolic health and disease. Nat. Rev. Microbiol..

[8] Freter R., Brickner H., Fekete J., Vickerman M.M., Carey K.E. (1983). Survival and implantation of *Escherichia coli* in the intestinal tract. Infect. Immun..

[9] Pereira F.C., Berry D. (2017). Microbial nutrient niches in the gut. Environ. Microbiol..

[10] Palmer C., Bik E.M., DiGiulio D.B., Relman D.A., Brown P.O. (2007). Development of the human infant intestinal microbiota. PLoS Biol..

[11] Kamada N., Chen G.Y., Inohara N., Nunez G. (2013). Control of pathogens and pathobionts by the gut microbiota. Nat. Immunol..

[12] Tagkopoulos I., Liu Y.-C., Tavazoie S. (2008). Predictive Behavior Within Microbial Genetic Networks. Science.

[13] Mitchell A., Romano G.H., Groisman B., Yona A., Dekel E., Kupiec M., Dahan O., Pilpel Y. (2009). Adaptive prediction of environmental changes by microorganisms. Nature.

[14] Kim M., Rai N., Zorraquino V., Tagkopoulos I. (2016). Multi-omics integration accurately predicts cellular state in unexplored conditions for *Escherichia coli*. Nat. Commun..

[15] Batt R.M., Peters T.J. (1976). Absorption of galactose by the rat small intestine in vivo: Proximal-distal kinetic gradients and a new method to express absorption per enterocyte. Clin. Sci. Mol. Med..

[16] Coombe N.B., Smith R.H. (1973). Absorption of glucose and galactose and digestion and absorption of lactose by the prepruminant calf. Br. J. Nutr..

[17] Ferraris R.P., Yasharpour S., Lloyd K.C., Mirzayan R., Diamond J.M. (1990). Luminal glucose concentrations in the gut under normal conditions. Am. J. Physiol..

[18] Thymann T., Moller H.K., Stoll B., Stoy A.C., Buddington R.K., Bering S.B., Jensen B.B., Olutoye O.O., Siggers R.H., Molbak L. (2009). Carbohydrate maldigestion induces necrotizing enterocolitis in preterm pigs. Am. J. Physiol. Gastrointest. Liver Physiol..

[19] Cleveland W.S. (1979). Robust Locally Weighted Regression and Smoothing Scatterplots. J. Am. Stat. Assoc..

[20] Malakar P., Venkatesh K.V. (2012). Effect of substrate and IPTG concentrations on the burden to growth of *Escherichia coli* on glycerol due to the expression of Lac proteins. Appl. Microbiol. Biotechnol..

[21] Shehata T.E., Marr A.G. (1971). Effect of Nutrient Concentration on the Growth of Escherichia coli. J. Bacteriol..

[22] Inada T., Kimata K., Aiba H. (1996). Mechanism responsible for glucose–lactose diauxie in Escherichia coli: Challenge to the cAMP model. Genes Cells.

[23] Kopp J., Slouka C., Ulonska S., Kager J., Fricke J., Spadiut O., Herwig C. (2017). Impact of Glycerol as Carbon Source onto Specific Sugar and Inducer Uptake Rates and Inclusion Body Productivity in *E. coli* BL21(DE3). Bioengineering.

[24] Lin E.C. (1976). Glycerol dissimilation and its regulation in bacteria. Annu. Rev. Microbiol..

[25] Cupples C.G., Miller J.H. (1989). A set of lacZ mutations in *Escherichia coli* that allow rapid detection of each of the six base substitutions. Proc. Natl. Acad. Sci. USA.

[26] Datsenko K.A., Wanner B.L. (2000). One-step inactivation of chromosomal genes in *Escherichia coli* K-12 using PCR products. Proc. Natl. Acad. Sci. USA.

[27] Reddy L., Poncet M., Self M.W., Peters J.C., Douw L., van Dellen E., Claus S., Reijneveld J.C., Baayen J.C., Roelfsema P.R. (2015). Learning of anticipatory responses in single neurons of the human medial temporal lobe. Nat. Commun..

[28] Rai N., Huynh L., Kim M., Tagkopoulos I. (2019). Population collapse and adaptive rescue during long-term chemostat fermentation. Biotechnol. Bioeng..

[29] Sharp S.J., Schaack J., Cooley L., Burke D.J., Soll D. (1985). Structure and transcription of eukaryotic tRNA genes. CRC Crit. Rev. Biochem..

[30] Zhou K., Zhou L., Lim Q.E., Zou R., Stephanopoulos G., Too H.-P. (2011). Novel reference genes for quantifying transcriptional responses of *Escherichia coli* to protein overexpression by quantitative PCR. BMC Mol. Biol..

[31] Rai N., Ferreiro A., Neckelmann A., Soon A., Yao A., Siegel J., Facciotti M.T., Tagkopoulos I. (2015). RiboTALE: A modular, inducible system for accurate gene expression control. Sci. Rep..

