# Supplementary material for: Understanding the Formation and Mechanism of Anticipatory Responses in Escherichia coli
Source: Int J Mol Sci. 2022 May 26;23(11):5985. doi: 10.3390/ijms23115985 (PMC9181292; doi:10.3390/ijms23115985)
Supplement: Supplementary file 1 [file ijms-23-05985-s001.zip › Supplementary file 1.pdf]

International Journal of *Molecular Sciences*

MDPI

Article

Understanding the Formation and Mechanism of Anticipatory Responses in *Escherichia coli*

Navneet Rai [1,2], Minseung Kim [1,2] and Ilias Tagkopoulos [1,2,3,*]

[1] UC Davis Genome Center, University of California-Davis, Davis, CA 95616, USA; nnrai@ucdavis.edu (N.R.); minseven77@gmail.com (M.K.)
[2] Department of Computer Science, University of California-Davis, Davis, CA 95616, USA
[3] USDA/NSF AI Institute for Next Generation Food Systems (AIFS), University of California, Davis, CA, 95616, USA
*Correspondence: itagkopoulos@ucdavis.edu

Supplementary information

1. Materials and methods.
2. Table 1. Primers used for qRT-PCR.
3. Table 2: List of primers used to integrate *gfp:kanR* cassette.
4. Table 3: List of primers used to repair the mutations.
5. Figure 1. Analysis of maximum growth rates of *E. coli* MG1655 in different carbon sources.
6. Figure 2. RT-PCR measurements of the fold change in the expression of selected target genes in *E. coli* MG1555 grown in M9 salt medium containing different carbon sources.
7. Figure 3. Characterization of MG1655 (*malP:gfp:kan*) strain. (A) Repression of the expression of *malP* in 10 mM Galactose. (B) Activation of the expression of *malP* in 10 mM Maltose.

Materials and methods

Transcriptome analysis of RNA-Seq experiments

A total of 43,931,731 single-end 50bp reads for 3 replicates of 3 samples was produced from *Illumina HiSeq* instrument. The fastq file produced from the machine was de-multiplexed into a separate file for each sample. Index sequences accompanying with reads were compared with pre-designed barcodes, allowing two base mismatches at most. Overall, samples had more than 4.3M raw reads. The low-quality of raw reads were trimmed using *Trimmomatic* (v0.30) with default settings. This procedure filtered out 4.8% of raw reads on average. The reads of the other samples were aligned on the most recent reference genome of *E. coli* MG1655 (NC_000913.3) by using *TopHat* (v2.0.10) coupled with *bowtie2* (v2.1.0) [4,5]. The overall alignment rate was 94.3%.

International Journal of
Molecular Sciences

DEG (Differentially Expressed Gene) analysis

For identifying differentially expressed genes, three methods including *Cuffdiff*, *edgeR*, and *DESeq* were applied and the lists of genes with statistical significance were merged afterwards. For *Cuffdiff* analysis, we in general followed the guideline introduced in [6]. FPKM normalization coupled with upper-quantile normalization was first measured using *Cufflink*. Then the normalized gene expression levels were used for DEG analysis using *Cuffmerge* and *Cuffdiff* with the default settings. For *edgeR* and *DESeq* analysis [7,8], we first removed gene having excessive zero counts across samples. All the downstream analysis was performed as suggested in the corresponding manuals. All the p-values computed from the methods above were adjusted using FDR (False Discovery Rate) following Benjamin-Hochberg procedure. Confidence threshold (α) was set to 0.1. Finally, the differential genes are determined by finding genes statistically significant by all three methods.

Figures

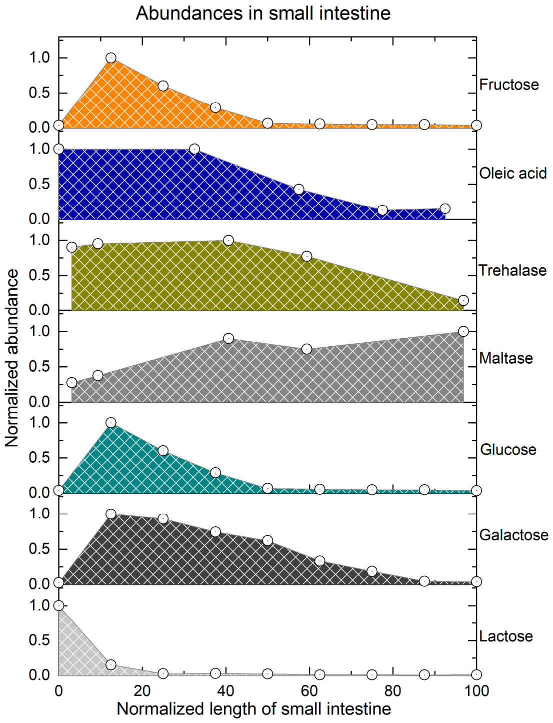

Figure S1. Abundances of different carbon sources in mammalian small intestine. Length of intestine is normalized from 0 to 100, while abundances of carbon sources are normalized from 0 to 1. Spatial concentration gradients of Maltase and Trehalase were used as proxies for the spatial concentration gradients of Maltose and Trehalose, respectively.

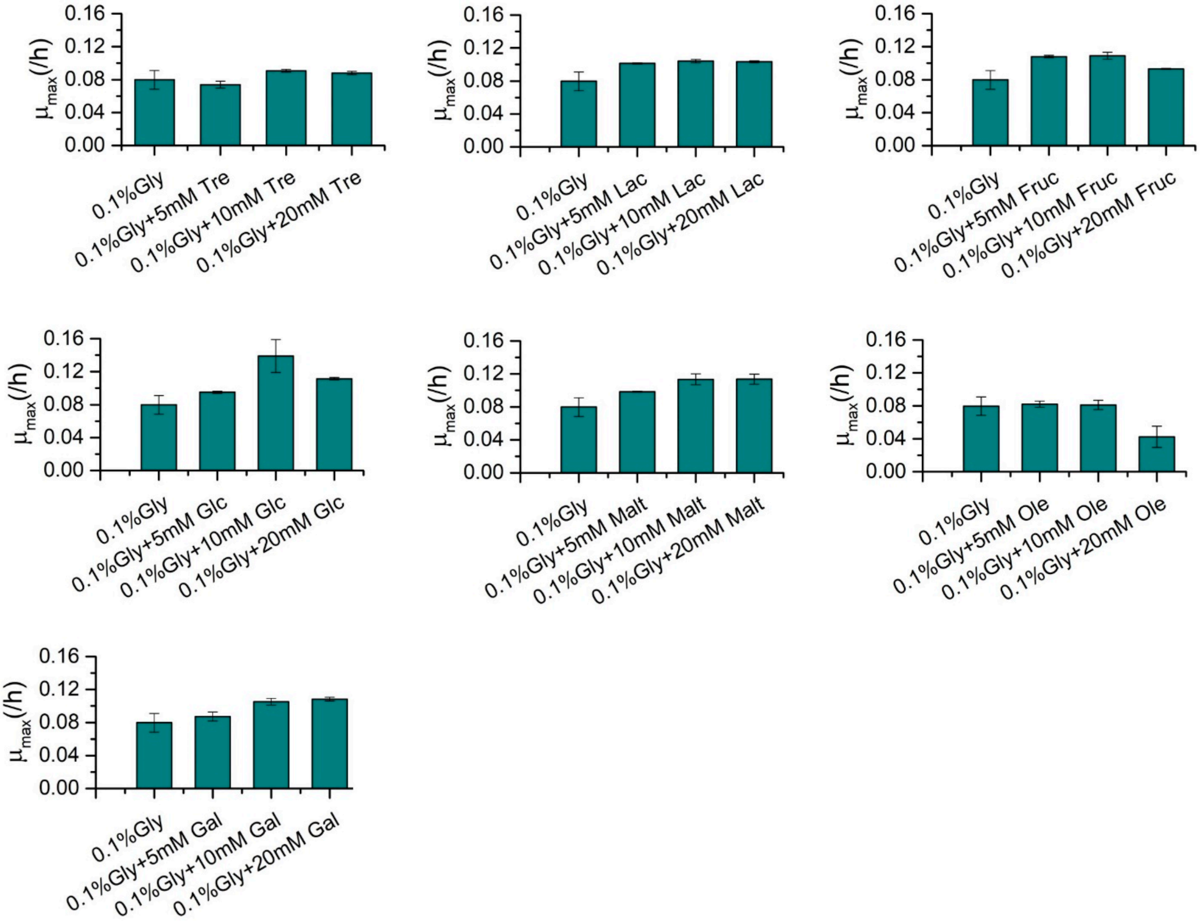

Figure S2. Analysis of maximum growth rates of *E. coli* MG1655 in different carbon sources. Gly; Glycerol, Tre; Trehalose, Lac; Lactose, Fruc; Fructose, Glc; Glucose, Malt; Maltose, Ole; Oleic acid, Gal; Galactose.

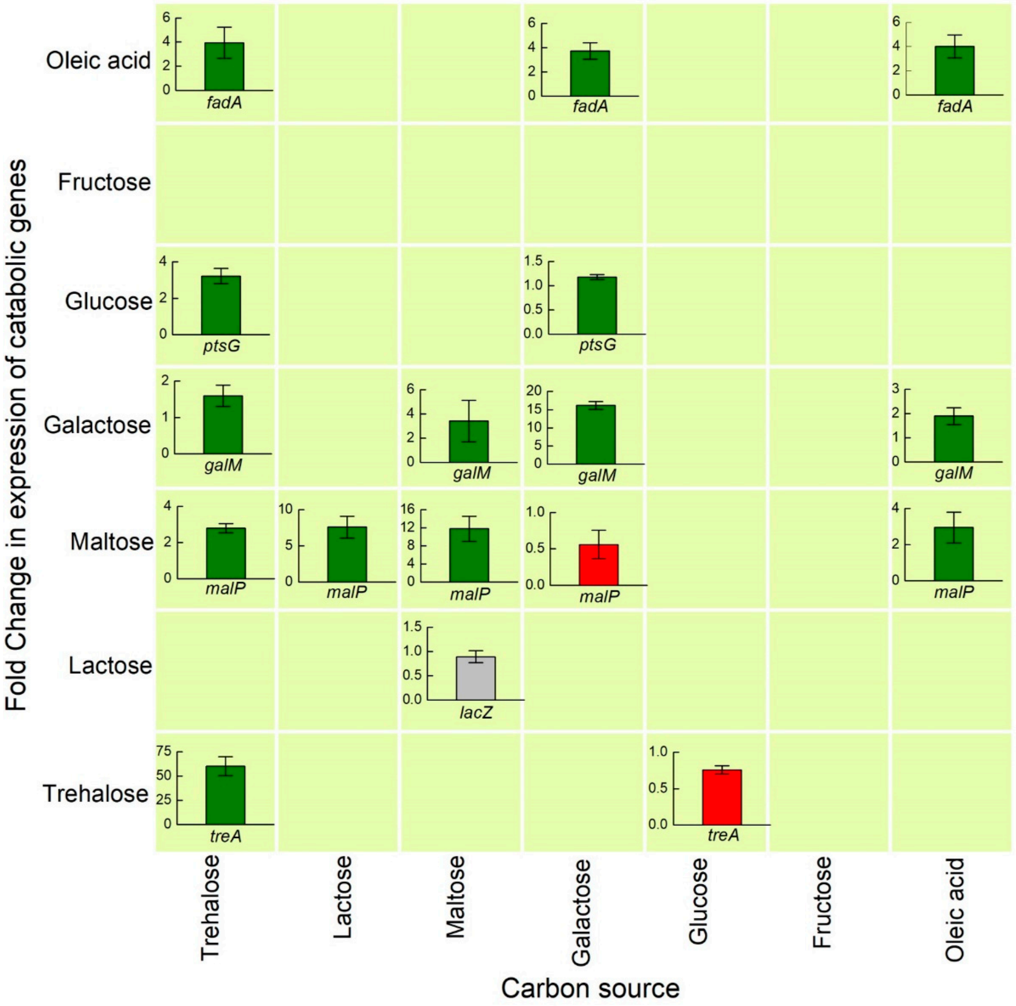

Figure S3. RT-PCR measurements of the fold change in the expression of selected target genes in *E. coli* MG1555 grown in M9 salt medium containing different carbon sources.

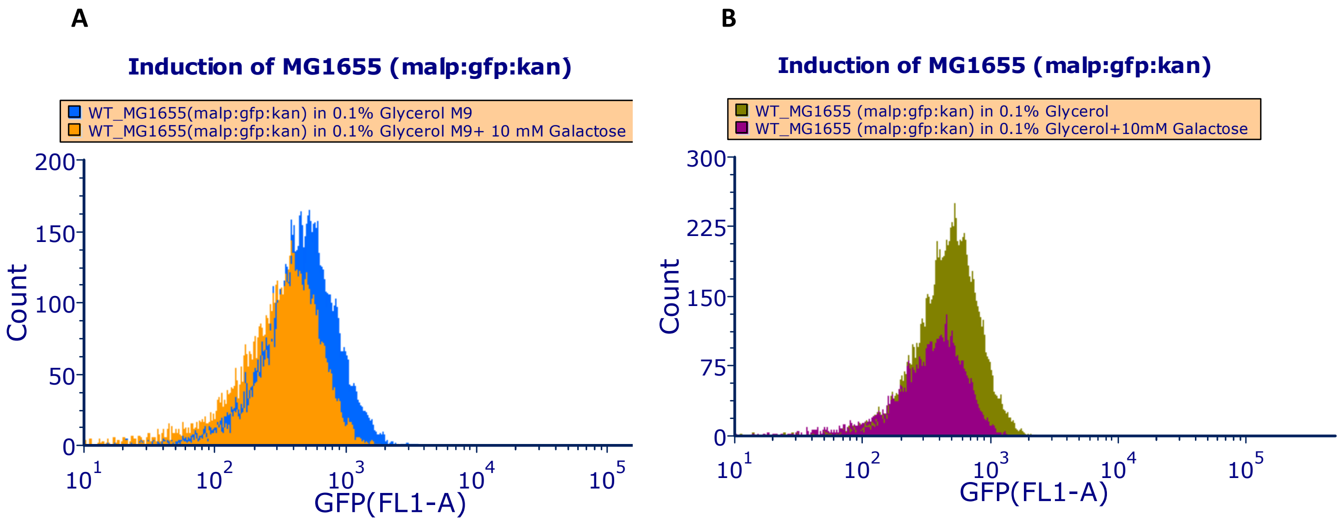

Figure S4. Characterization of MING (MG1655 with *malP:gfp:kan*) strain. (**A**) Repression of the expression of *malP* in 10 mM Galactose. (**B**) Activation of the expression of *malP* in 10 mM Maltose.

Tables

Table S1. Primers used for qRT-PCR

S. No.	Gene	Primer	Sequence (5→3)
1	*treB*	Forward	TTGATTAGCGGCGGTTTGATCCTCGG
		Reverse	TGACCAGCAAATACCGACCGGCAG
2	*ptsG*	Forward	GTTCCGTCTTTGCAAACATGCCACTG
		Reverse	AGTACGCCAGTATCCGCCAGGTG
3	*fadA*	Forward	AGAAGTACCACACTCTGTCCCGGC
		Reverse	CTTTGGCGACATTGCGGCTCAGG
4	*galM*	Forward	TGGTCAGCCGTACCGACTGTTAACTTTG
		Reverse	TAACGACCAATAGAGGCCCCCAGAAACG
5	*ihfB*	Forward	AATCGCACATTCCCGCCAAGACG
		Reverse	TCCAGTTCTACTTTATCGCCAGTCTTCGG
6	*lacZ*	Forward	TACCCAACTTAATCGCCTTGCAGCAC
		Reverse	CGGCCTCAGGAAGATCGCACTC
7	*malP*	Forward	ATGACATCAATCTGACGGACCTGCTG
		Reverse	AGTCATCCGGCGCTTCAACCTGTTTGC

International Journal of
Molecular Sciences

MDPI

Table S2 List of primers used to integrate *gfp:kanR* cassette

S. No.	Primer	Sequence (5→3)
1	Gfpkan_rv1	TCCCGTTGAATATGGCTCATTCTAGATTTCTCCTCTTTAATCTCTAGTAT TATTTGTATAGTTCATCCATGCCATGTGTAATCCCAG
2	Gfp_in_fw	AGCTCGGATCGCTCTATTCGCGATTATCAGGCTCGTATCTGGCAGGCA AAACGCTAATACTAGAGATTAAAGAGGAGAAATACTAGATGCGTAAA GG
3	Kan_in_fw	TGGGGCTAATCCCCGCCGCCAGCGCGGCATTATCCAGACGTTTGCTTTC CATCGAGCTTCCTTAGAAAAACTCATCGAGCATCAAATGAAACTGC
4	Kan_gfp_rv	CTGGGATTACACATGGCATGGATGAACTATACAAATAATACTAGAGAT TAAAGAGGAGAAATCTAGAATGAGCCATATTCAACGGGAAACGTC

Table S3. List of primers used to repair the mutations

S. No.	Primer	Sequence (5→3)
1	SP7.2_Fw	GAGAAAGTGGGCAACCTGGCGTTCCTCGACGTGACGGAACAGGCTTA CCCGTCTTACTGTCC
2	SP7.4_Fw	GTACTACATCGTTAAAGGCTCTGTGGCAGTGCTGATCAAACACAGGCT TACCCGTCTTACTGTCC
3	MT1S_Fw	TTCAGACACATAAAAAAACGTCATCGCTTGCATTAGAAAGGTTTCTGG CCTACAGGCTTACCCGTCTTACTGTCC
4	SPU_Rv	GGGGGAAACAAAATGGCGCGCTACCAGGTAACGCGCCACTCCGACGT CGGTTCGTAAGCTGTAATGCAAGTAGC
5	MT1S_Rv	TTGAATGATGCAGAGATGTAAGCCGGATCTGGCGCGTTATCCGGCTAA ACTCGGTTCGTAAGCTGTAATGCAAGTAGC
6	Crp7.4_Fw	GTACTACATCGTTAAAGGCTCTGTGGCAGTGCTGATCAAACACGAAGA GGGTAAAGAAATGATCCTCTCCTATCTG
7	Crp7.2_Fw	GAGAAAGTGGGCAACCTGGCGTTCCTCGACGTGACGGACCGCATTGC ACAGACTCTGCTG
8	Crpc_Rv	CTTTAATCTCTAGATTTAACGAGTGCCGTAAACGACGATGGTTTTAC
9	Cm_Fw	ACTCGTTAAATCTAGAGATTAAAGAGGAGAAATACTAGATGGAGAAA AAAATCACGGGATATACCACCG
10	Cm_Rv	GGGGGAAACAAAATGGCGCGCTACCAGGTAACGCGCCACTCCGACGG GATTACGCCCCGCCCTGCCACTC
11	MalTP1_Fw	TTCAGACACATAAAAAAACGTCATCGCTTGCATTAGAAAGGTTTCTGG CCTACCTTATAACCATTAATTACGAAGCGCAAAAAAAATAATATTTCC TCAT
12	MalT_Rvn	TTAATCTCTAGATTTACACGCCGTACCCCATCATC
13	MT1C_Rv	TTGAATGATGCAGAGATGTAAGCCGGATCTGGCGCGTTATCCGGCTAA ACTTACGCCCCGCCCTGCCACTC
